# A generative adversarial network to improve integrated mode proton imaging resolution using paired proton–carbon data

**DOI:** 10.1002/mp.18081

**Published:** 2025-09-09

**Authors:** Mikaël Simard, Ryan Fullarton, Lennart Volz, Christoph Schuy, Savanna Chung, Colin Baker, Christian Graeff, Charles‐Antoine Collins Fekete

**Affiliations:** ^1^ Department of Medical Physics and Biomedical Engineering University College London London UK; ^2^ Biophysics GSI Helmholtz Centre for Heavy Ion Research GmbH Darmstadt Germany; ^3^ Department of Radiotherapy Physics University College London Hospital, NHS Foundation Trust London UK

**Keywords:** generative adversarial network, image‐to‐image translation, ion beam therapy, ion imaging, ion radiography, super‐resolution imaging

## Abstract

**Background:**

Integrated mode proton imaging is a clinically accessible method for proton radiographs (pRads), but its spatial resolution is limited by multiple Coulomb scattering (MCS). As the amplitude of MCS decreases with increasing particle charge, heavier ions such as carbon ions produce radiographs with better resolution (cRads). Improving image resolution of pRads may thus be achieved by transferring individual proton pencil beam images to the equivalent carbon ion data using a trained image translation network. The approach can be interpreted as applying a data‐driven deconvolution operation with a spatially variant point spread function.

**Purpose:**

Propose a deep learning framework based on paired proton–carbon data to increase the resolution of integrated mode pRads.

**Methods:**

A conditional generative adversarial network, Proton2Carbon, was developed to translate proton pencil beam images into synthetic carbon ion beam images. The model was trained on 547 224 paired proton–carbon images acquired with a scintillation detector at the Marburg Ion Therapy Centre. Image reconstruction was performed using a 2D lateral method, and the model was evaluated on internal and external datasets for spatial resolution, using custom 3D‐printed line pair modules.

**Results:**

The Proton2Carbon model improved the spatial resolution of pRads from 1.7 to 2.7 lp/cm on internal data and to 2.3 lp/cm on external data, demonstrating generalizability. Water equivalent thickness accuracy remained consistent with pRads and cRads. Evaluation on an anthropomorphic head phantom showed enhanced structural clarity, though some increased noise was observed.

**Conclusions:**

This study demonstrates that deep learning can enhance pRad image quality by leveraging paired proton–carbon data. Proton2Carbon can be integrated into existing imaging workflows to improve clinical and research applications of proton radiography. To facilitate further research, the full dataset used to train Proton2Carbon is publicly released and available at https://zenodo.org/records/14945165.

## 1 INTRODUCTION

Integrated mode proton imaging, where signals are acquired from individual pencil beams and analyzed independently, is a clinically accessible mode for proton radiographs (pRads).^1^ However, it suffers from limited spatial resolution compared to single‐event imaging due to multiple Coulomb scattering (MCS). The spatial resolution of integrated mode imaging can be improved with advanced‐physics‐based models,^2^ or via the development of deconvolution kernels tailored to specific detectors and ion beam shapes.^3^, ^4^ In this study, a deep learning framework based on an image translation task to improve the image quality of pRads is proposed.

The amplitude of MCS in beams of various ion species decreases with increasing particle mass, when compared at a constant range across ion beams.^5^ This is generally associated with improved image resolution for heavier ion species, as demonstrated for single‐event^6^ and integrated mode^7^ ion imaging. While machine learning methods have been proposed to improve the quality of medical imaging for most modalities, there are no available approaches yet in ion imaging due to the lack of training data. However, for integrated mode imaging, there are typically hundreds or thousands of individual pencil information recorded for the generation of a single radiograph. If ion radiographs of the same object are acquired with different ion species, as achieved in earlier work,^7^, ^8^ a large dataset of paired pencil beam data is accessible. In this context, this work proposes a new method to improve resolution of integrated mode pRads, which translates each integrated measurement from an individual pencil beam acquired with proton beams, suffering from high MCS, to the equivalent measurement acquired with a carbon ion beam. This image translation task is achieved using a trained deep learning framework. The approach is analogous to learning a deconvolution operation with a spatially variant point spread function.

This work introduces the Proton2Carbon model, a generative adversarial network (GAN) that converts images of individual proton beams to carbon ion beams to improve the image quality of pRads. The images are obtained using a detector combining a scintillator and CCD cameras and produces state‐of‐the‐art integrated mode ion radiographs, as described in previous studies.^2^, ^8^, ^9^ The performance of the Proton2Carbon model is evaluated for spatial resolution improvement on both internal and external datasets. Training was conducted on a dataset of 547 224 paired proton–carbon images, termed the Proton2Carbon dataset, which is made publicly available to encourage further research in machine learning for ion radiography enhancement.[Supplementary-material mp18081-supl-0001]

## 2 METHODS

**FIGURE 1:**
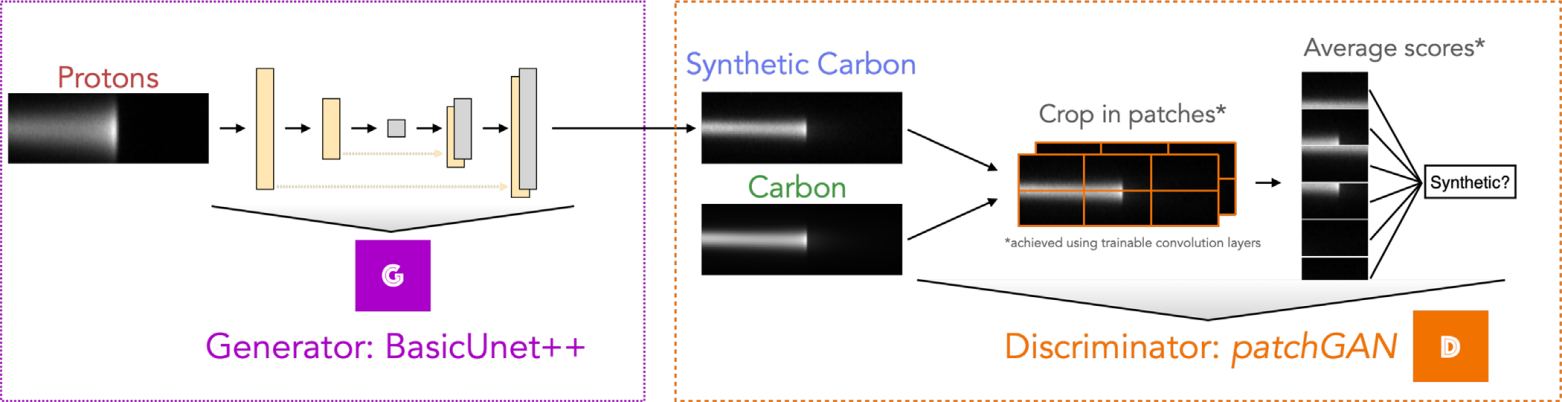
Architecture of the Proton2Carbon model based on the pix2pix architecture.^10^

We first introduce the dataset in Section 2.1, and the Proton2Carbon model architecture and training are in Section 2.2 and Figure 1. The methods used for radiograph reconstruction and evaluation are discussed in Section 2.3.

### 2.1 Data acquisition

**FIGURE 2:**
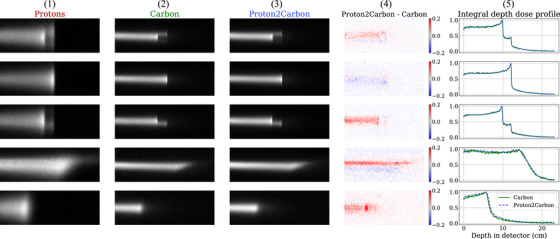
Performance of the Proton2Carbon model on 5 exemplar pencil beams with various levels of range mixing. From left to right, the columns show the raw (1) proton and (2) carbon images, (3) the synthetic carbon image, (4) the difference between true and synthetic carbon, and (5) the integrated pencil beam image over the lateral dimension to create 1D signals analogous to a Bragg curve, for true and synthetic carbon images. The dynamic range in the first three columns is 0–1.

The Proton2Carbon dataset consists of 547 224 pairs of proton and carbon ion pencil beam images obtained using a scintillation detector. Each image represents a pencil beam passing through an object positioned between the source and the detector at a specific location within the imaging field of view. Examples are shown in Figure 2, including pristine Bragg curves as well as beams exhibiting range mixing effects due to heterogeneous geometries. A complete scan of an object involves acquiring N pencil beam images that adequately sample the object. For this study, each scan was performed using a pencil beam scanning approach covering a 151 × 151 ${\mathrm{mm}}^{2}$ field of view (FOV) with a beam spacing of 1 mm, resulting in 22 801 pencil beam images per camera view.

Each proton image is fully registered with its corresponding carbon image. The dataset is a subset of a series of experiments conducted on a Siemens synchrotron at the Marburg Ion Therapy Centre, designed to compare the image quality and real‐time tracking capabilities of pRads and carbon ion radiographs (cRads) using multiple phantoms.^7^, ^8^ It includes 12 scans of different phantoms and geometries, detailed in Section 1 of the supplementary material. The scanned phantoms include a Gammex phantom, an anthropomorphic head phantom, custom 3D‐printed line pair modules, and custom 3D‐printed low‐contrast modules. The dataset is publicly available at https://zenodo.org/records/14945165 , with additional details on image format and organization provided in Section 2 of the supplementary material.

To assess the generalizability of the Proton2Carbon model, an external test set was created by imaging the 3D‐printed line pair modules with the same detector setup at a different accelerator (Varian ProBeam cyclotron at University College London Hospitals (UCLH)) using a 151 × 151 ${\mathrm{mm}}^{2}$ FOV, a beam spacing of 1 mm, and an energy of 200 MeV. The spot size of the beam at 200 MeV is different at UCLH (≈7‐mm FWHM compared to 9.4 mm at the Marburg Ion Therapy Centre).

### 2.2 Model architecture and training

A conditional GAN was trained based on the pix2pix architecture,^10^ which has demonstrated high performance in image translation tasks, particularly in preserving the larger spatial frequencies of the target image. The architecture of the Proton2Carbon network is illustrated in Figure 1. The network includes a generator designed to create synthetic pencil beam carbon images from proton pencil beam images. The generator is a nested Unet++ network, proposed by Zhou et al.^11^ The discriminator follows the default patchGAN approach proposed for pix2pix,^10^ providing a receptive field of approximately 70 × 70 pixels 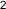. All training hyperparameters and details are provided in Section 3 of the supplementary materials.

**FIGURE 3:**
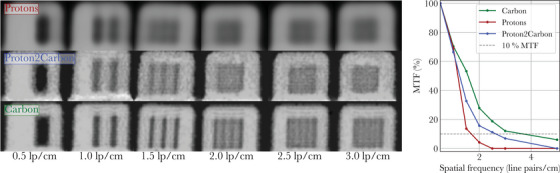
Ion radiographs of line pair modules and associated modulation transfer functions (MTFs). The line pair modules were scanned at the same institution where the training data for the Proton2Carbon model were acquired.

For any result shown on a given scanned object, it is assumed that the model was trained without any data from this scanned object. To achieve this, different models were trained while excluding specific scans from each training set. For instance, to generate the results in Figure 3 on the line pair modules, all five scans containing spatial resolution modules were excluded and left as a test set, while the remaining seven scans were used for training and validation. The exact splits for each model are detailed in Section 3.2 of the supplementary material.

### 2.3 Image reconstruction and performance evaluation

To convert a dataset of N pencil beam images into an ion radiograph, the 2D lateral approach introduced by Simard et al.^2^ is used. In this framework, pRads and cRads for each of the 12 scans can be reconstructed from the proton and carbon pencil beam images of the corresponding dataset introduced in Section 2.1. Similarly, synthetic cRads can be generated by taking a set of N proton pencil beam images, producing the corresponding N synthetic carbon ion pencil beam images using the Proton2Carbon model, and then applying the 2D lateral reconstruction approach to the set of synthetic carbon ion pencil beam images.

**FIGURE 4:**
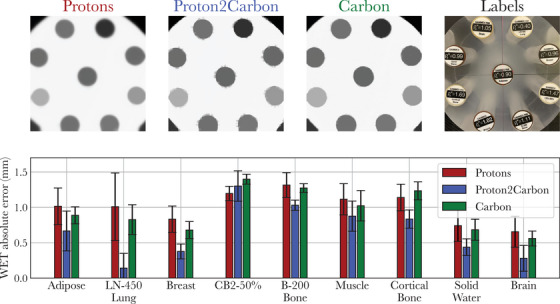
Ion radiographs of the Gammex phantom and water equivalent thickness (WET) absolute error for each insert.

**FIGURE 5:**
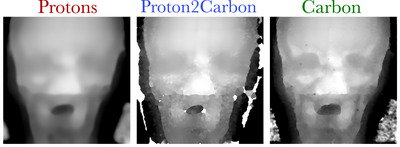
Ion radiographs of an anthropomorphic head phantom.

**FIGURE 6:**
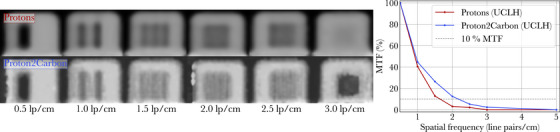
Ion radiographs of line pairs modules and associated modulation transfer functions (MTFs). The line pair modules were scanned at a different institution as the training data used for the Proton2Carbon model.

Figure 2 illustrates the performance of Proton2Carbon in generating synthetic carbon ion pencil beam images. The image quality of synthetic cRads is analyzed in Figures 3, 4, 5, 6. For spatial resolution, Figures 3 and 6 present modulation transfer function (MTF) calculations for a range of custom 3D‐printed line pair inserts, following the methodology described by Simard et al.^7^ The resolution is estimated as the spatial frequency at which the MTF reaches 10%. Additionally, the quantitative accuracy of the water equivalent thickness (WET) is evaluated in Figure 4 using the Gammex phantom by calculating the WET absolute error across all nine material inserts. The WET absolute error is determined by averaging the WET value within a circular region covering 80% of each insert's size. Finally, image quality is also assessed using an anthropomorphic head phantom (Figure 5). Image similarity was assessed visually and quantitatively via the calculation of the structural similarity index measure (SSIM). The average WET difference per pixel is also reported between pRads and cRads, as well as between synthetic cRads and cRads.

## 3 RESULTS

Figure 2 presents the image translation capabilities of the Proton2Carbon network for individual pencil beam images.

Figure 3 illustrates relevant ion radiographs (pRads, synthetic cRads from proton data using the Proton2Carbon model, and cRads) of six custom 3D‐printed line pair modules scanned at the Marburg Ion Therapy Centre, and the associated MTFs. Figure 4 presents similar ion radiographs for the Gammex phantom, along with the WET absolute error for each insert.

Quantitative metrics related to Figures 3 (spatial resolution) and 4 (WET quantitative accuracy) are reported in Table 1, for pRads, synthetic cRads, and cRads.

**TABLE 1:** Image quality metrics for proton radiographs, synthetic carbon ion radiographs generated with the Proton2Carbon model, and carbon ion radiographs. The data used to train the Proton2Carbon model is scanned at the same institution as the objects scanned to obtain the above quantitative metrics.

Image quality metric	pRads	Synthetic cRads	cRads
Resolution (lp/cm)	1.7	2.7	3.7
WET accuracy (mm)	1.0	0.8	1.0

The image quality of ion radiographs can be assessed qualitatively in a realistic anthropomorphic head in Figure 5.

For the anthropomorphic head phantom, synthetic cRads are structurally highly similar to cRads, with a structural similarity index of 0.97, as opposed to 0.92 between pRads and cRads. Furthermore, the mean WET difference per pixel between synthetic cRads and cRads is 3.4 mm, compared to 6.5 mm for pRads against cRads.

Finally, spatial resolution was assessed on data scanned at a different institution (UCLH) from the training data. Proton and synthetic carbon ion radiographs are presented in Figure 6, similarly to Figure 3. Spatial resolution increased from 1.7 lp/cm with pRads to 2.3 lp/cm with synthetic cRads.

## 4 DISCUSSION AND CONCLUSIONS

This work demonstrates that the image quality of integrated mode proton radiographs can be improved with the Proton2Carbon deep learning network, a GAN trained to create synthetic carbon ion pencil beam images from proton pencil beam images.

Based on Figure 2, the network is usually able to reconstruct the 2D carbon pencil beam data from proton data with high fidelity. Most of the errors (fourth column of Figure 2) are noise, as the smooth structure of Proton2Carbon images cannot reproduce the noisy behavior of carbon images. However, it is observed that some images create artificial structures leading to a shift in the apparent range of the beam, such as the last example in Figure 2. This is consistent with the fact that the pix2pix architecture is found to introduce artifacts in regions where the input image is sparse,^10^ such as towards the distal edge of a pencil beam. Artifacts have been mitigated via the use of multiple regularization techniques (Section 3, supplementary material), but may benefit from further improvement. A possible avenue to limit such artifacts may be to introduce further regularization based on the expected task downstream from the image translation task, that is, the localization of one or multiple Bragg peaks in the 2D images. This may be difficult to implement due to the nonconvexity of the associated cost function, and we leave this avenue to future work.

Generally, the image quality of synthetic cRads lies between that of pRads and cRads. Table 1 shows that the Proton2Carbon model improves spatial resolution of pRads (from 1.7 to 2.7 lp/cm), although it does not fully reach the resolution of true cRads (3.7 lp/cm). These results are based on scans acquired at the same institution where the Proton2Carbon training data were collected. When considering data from another institution (Figure 6), the spatial resolution of pRads is also found to increase (from 1.7 to 2.3 lp/cm), which suggests good generalization capabilities for Proton2Carbon. The resolution achieved on the internal test set (2.7 lp/cm) exceeds that on the external test set (2.3 lp/cm), despite the use of the same detector. This suggests that the network may have learned institution‐specific features from the MIT dataset, leading to a slight degradation in performance when applied to data from a different institution. We attribute this discrepancy primarily to differences in accelerator technology (cyclotron at UCLH against synchrotron at MIT) and different beam sizes at 200 MeV, as described in Section 2.1. In future work, incorporating training data from multiple institutions may help mitigate this domain shift and improve generalization across sites.

The improvements in spatial resolution come without compromising WET quantitative accuracy. As shown in Table 1, synthetic cRads achieve a similar WET accuracy to both pRads and cRads on the Gammex phantom. Experiments on the anthropomorphic phantom show that synthetic cRads generally appear sharper than pRads (Figure 5). The improved SSIM and reduced mean WET difference per pixel suggest that the Proton2Carbon network improves the structural and quantitative similarity of pRads with cRads. Nonetheless, one drawback is that images generally exhibit increased noise, which is the main limitation of the proposed network. This may be explained, as previously discussed, by an imperfect image translation of individual pencil beams, especially in the presence of strong range mixing or scattering, which is noticeable in the absolute difference images of Figure 2. While the Proton2Carbon network appears successful at generating synthetic integral depth dose profiles, the spatial localization of beams in the lateral dimension (i.e., peak finding) can be erroneous, which can lead to noise in reconstructed images when using reconstruction methods that harness the 2D information of the images such as the one used in this work.^2^ To address the noise issue, future work will focus on expanding the dataset to include additional phantoms, institutions, energy levels, and scattering conditions. Another limitation is that the model is currently restricted to images acquired using a scintillator and CCD cameras. However, the underlying approach could be extended to other integrated‐mode ion imaging frameworks.

Finally, we note image quality artifacts at some material interfaces in the carbon and synthetic carbon images of Figure 4, where transitions between materials appear speckled rather than smooth, unlike in the proton images. This effect arises from limitations in the 2D reconstruction framework,^2^ particularly when handling narrow beams at interfaces with high WET gradients. The reconstruction framework estimates WET independently in the lateral and top views via a peakfinding routine (e.g., fig. 2b,c in Simard et al.^2^), which lacks robustness for narrow beams such as those shown in the third row of Figure 2. In some cases, each view detects only the Bragg peak of a different material. The final WET, computed as the average of both views, is thus incorrect at the interface, resulting in the observed artifacts. This effect is primarily observed with carbon ion beams, due to their narrow lateral spread. While the Proton2Carbon network slightly amplifies this issue, it is expected that improvements to the peakfinding routine, such as detecting and correcting large identified WET between views, could mitigate these artifacts. Such refinements of edge cases are left to future work.

This study serves as a proof of concept that pRad image quality can be enhanced using a conditional GAN trained on matched proton–carbon data. Training the network at the raw data level (i.e., individual pencil beam images) enables the creation of a large dataset, facilitating the development of a model with robust generalization capabilities. The Proton2Carbon network can be incorporated in any image acquisition/reconstruction pipeline to augment the image quality of pRads.

## CONFLICT OF INTEREST STATEMENT

The authors declare no conflicts of interest.

## Supporting information

Supporting Information
