# Supplementary material for: A generative adversarial network to improve integrated mode proton imaging resolution using paired proton–carbon data
Source: Med Phys. 2025 Sep 9;52(9):e18081. doi: 10.1002/mp.18081 (PMC12421214; doi:10.1002/mp.18081)
Supplement: Supplementary file 1 — Supporting Information [file MP-52-0-s001.pdf]

Supporting material for: A generative adversarial network to improve integrated mode proton imaging resolution using paired proton-carbon data

Section 1: image acquisition parameters and geometries for each scan

The experimental setup for image acquisition is shown in figure 1 of Simard *et al* [1], while details on the scintillator, including the camera positioning, are detailed in figure 1 of Fullarton *et al* [2].

All scans are performed with a 15.1x15.1 cm^2 FOV with 1 mm beam spacing, for a total of 151x151 = 22,801 pencil beams per camera view. There are two camera views (lateral to the scintillator, and top view of the scintillator), for a total of 45,602 images per geometry, per ion species. Factoring the 12 scans, this results in a total of **547,224 paired proton and carbon images**.

Table 1 lists the scans performed to generate the Proton2Carbon database. For each scan, the object is positioned between the beam and scintillator. Proton and carbon data are then sequentially acquired, such that each pencil beam data is fully registered between ion species.

Scans using a custom 3D printed phantom (#3, 8-12) also included an additional 10 cm of solid water to increase scattering. Additional details on the phantoms can be found in Simard *et al* [1].

Table 1. List of the 12 scans performed to generate the Proton2Carbon dataset.

Scan #	Proton energy (MeV/u)	Carbon energy (MeV/u)	Geometry/phantom
1	180	344	No object
2	200	390	No object
3	180	344	Custom 3D printed low-contrast module
4	180	344	Gammex
5	180	344	Anthropomorphic head phantom, front view

6	200	390	Anthropomorphic head phantom, front view
7	180	344	Anthropomorphic head phantom, lateral view
8	180	344	Custom 3D printed spatial resolution modules, configuration 1: 1.5, 1, 0.5 line pairs / cm.
9	180	344	Custom 3D printed spatial resolution modules, configuration 2: 0.5, 1.5, 1 line pairs / cm.
10	180	344	Custom 3D printed spatial resolution modules, configuration 3: 1, 0.5, 1.5 line pairs / cm.
11	180	344	Custom 3D printed spatial resolution modules, configuration 4: 2, 2.5, 3 line pairs / cm.
12	180	344	Custom 3D printed spatial resolution modules, configuration 5: 3, 2, 2.5 line pairs / cm.

Relevant images of the geometries/phantoms are presented below.

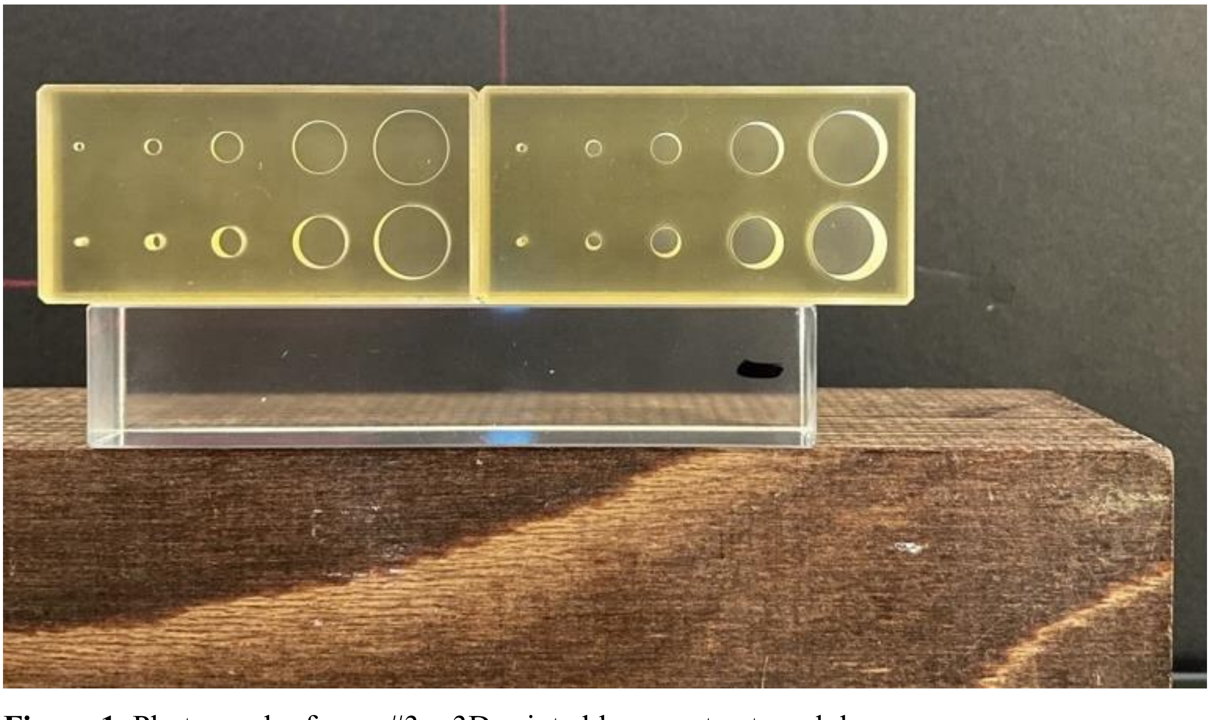

Figure 1. Photograph of scan #3 – 3D printed low-contrast module

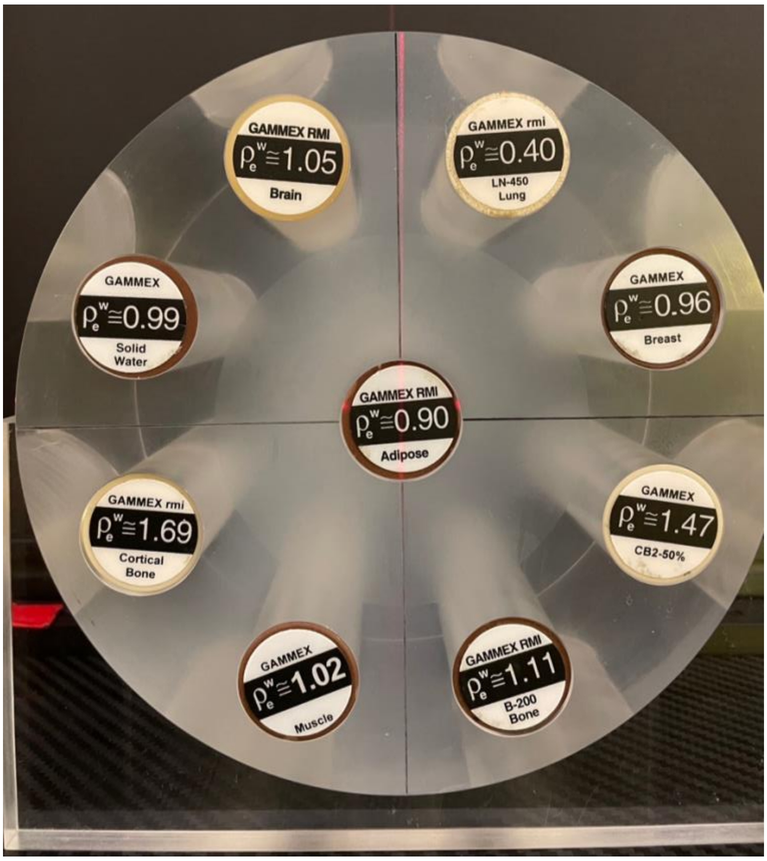

Figure 2. Photograph of scan #4 – Gammex phantom with inserts

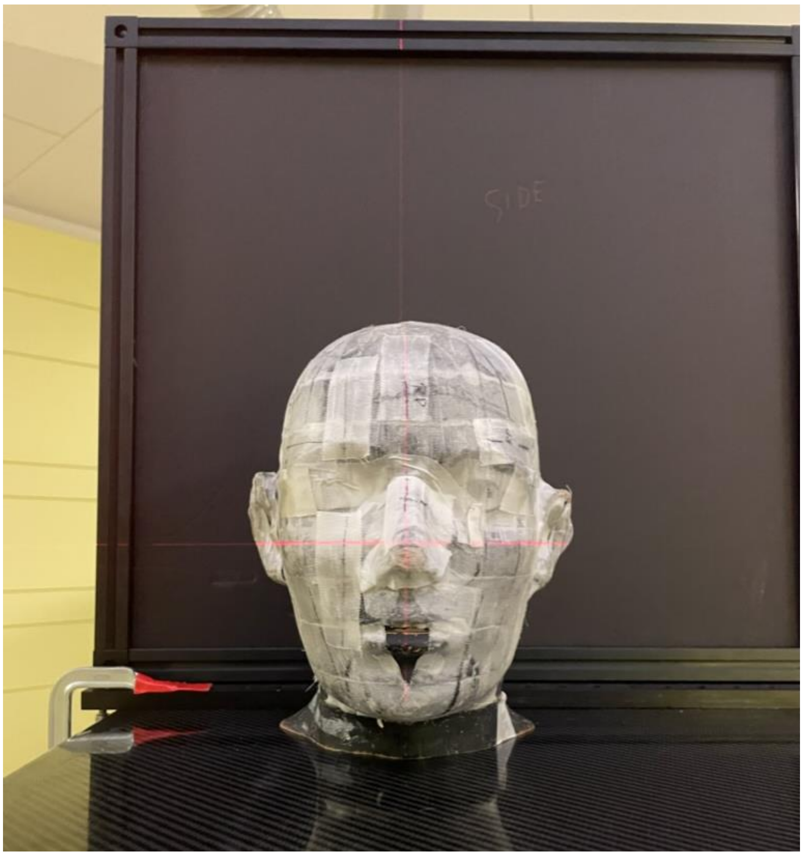

Figure 3. Photograph of scans #5 and #6 – anthropomorphic head phantom, front view

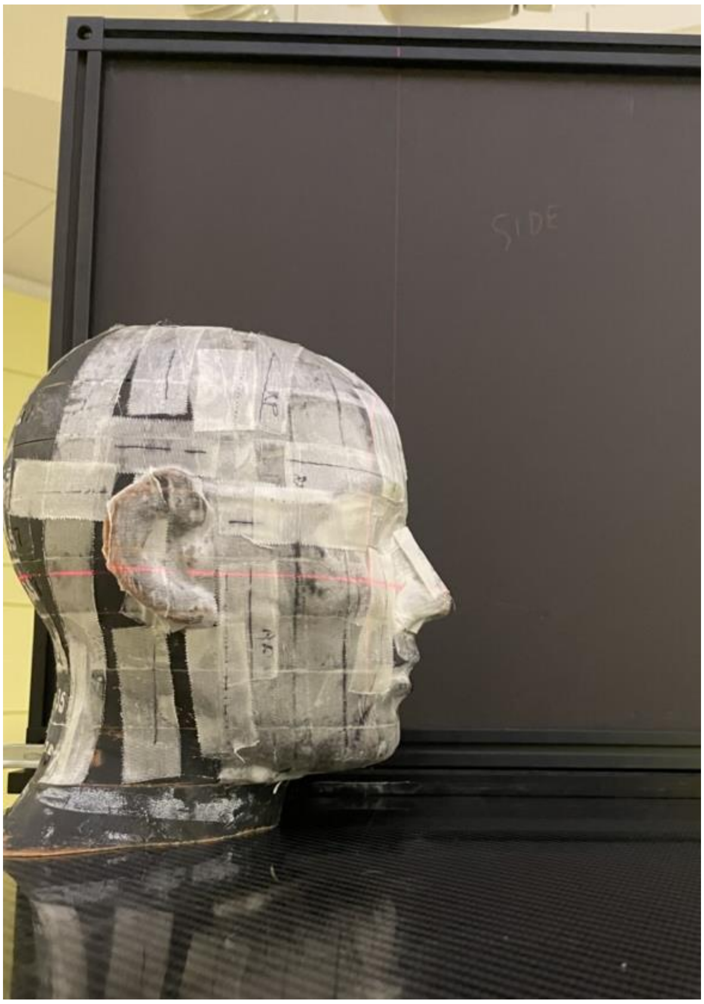

Figure 4. Photograph of scan #7 – anthropomorphic head phantom, lateral view

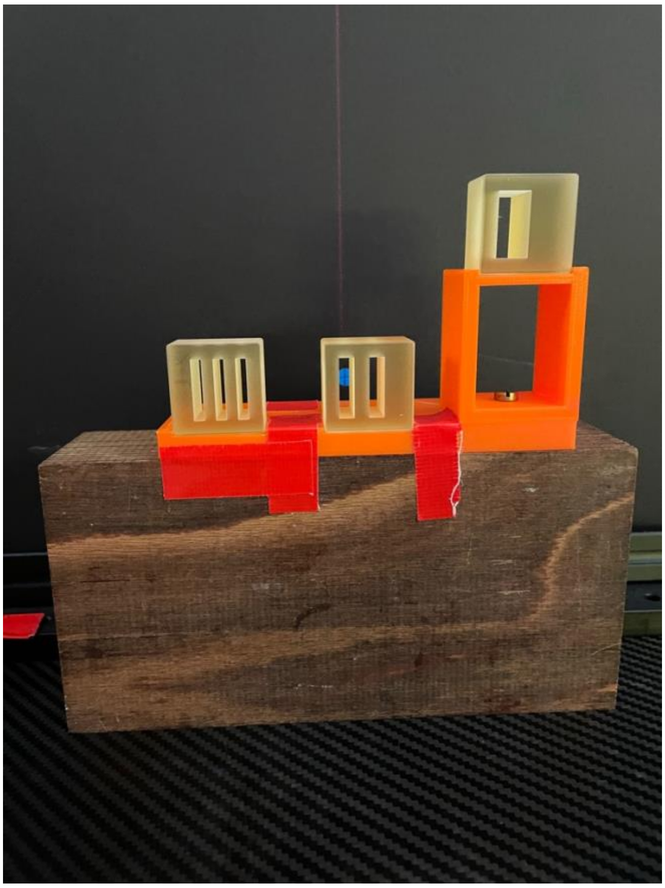

Figure 5. Photograph of scan #8 – Custom 3D printed spatial resolution modules, configuration 1, with (from left to right) the 1.5, 1 and 0.5 line pairs / cm modules.

Figure 5 illustrates the first configuration of 3D printed spatial resolution modules. To generate the five configurations, we used various permutations of six 3D printed line pair modules in the same spatial organisation as shown in figure 5. The 6 3D printed line pair modules are the 6 bottom ones shown in figure 1(d) of Simard *et al* [1]. They respectively represent spatial resolutions of 0.5, 1, 1.5, 2, 2.5, and 3 line pairs / cm. Figure 5 shows, from left to right, the first three: 1.5, 1 and 0.5 line pairs / cm. The other configurations are outlined in table 1.

Section 2: Proton2Carbon dataset descriptor

Each of the 547,224 images is a (W, H) = (608, 96) pixels .png image containing a (pulled back) pencil beam, with various levels of range mixing. The camera pixel size is approximately 0.41 mm; each image covers a physical field of view of approximately 250×40 mm^2. While the scintillator acquires raw images of size (608, 488), each image was cropped to a 4 cm region

around the centre of the pencil beam in the height dimension to reduce the size of the images and network.

For users interested in image reconstruction, we provide the coordinate of the crop for each image in the `cropped_coordinates.csv` file such that the full (608, 488) images can be recreated. To recreate the full image, assuming that `cropped_image` is the (608, 96) image, one can do

```
full_image = np.zeros((608, 488))
full_image[yi:yf, xi:xf] = cropped_image
```

Where `xi, xf, yi, yf` are tabulated in the `.csv` file.

The dataset contains 12 zipped folders, one for each of the 12 scans introduced in section 1. The data structure is:

./<scan_number>/<ion_species>/<camera_view>_image_#.png

Where <scan_number> ∈ {scan_1, …, scan_12} and correspond to the 12 geometries introduced in section 1, <ion_species> ∈ {protons, carbon}, <camera_view> ∈ {lateral, top}, and # represents the number of the image, ranging from 1 to 22,801 for each combination of scan, ion species and camera view. The matching proton and carbon ion images will have the same filename except the <ion_species>.

The dataset is publicly available at https://zenodo.org/records/14945165.

Section 3: Proton2Carbon model training and hyperparameters

3.1 Model hyperparameters

All trained models follow the architecture of the pix2pix model [3]. For all models, we used a batch size of 16, and 50 epochs. For optimisation, the Adam algorithm was used with $\beta_1, \beta_2 = 0.5, 0.999$, and a weight decay of 5×10^{-5} was used. Learning rates for the generator and discriminator of respectively 10^{-4} and 10^{-5}. An ℓ_1 weight decay of 100

was used for the generator, and label smoothing with a weight of 0.03 was used. A cosine annealing scheduler was used for the learning rate.

The generator is the basic Unet++ from MONAI and uses a drop rate of 0.2, leakyReLU activations, and encoding features of size [32, 32, 64, 128, 256, 32]. The discriminator, the patchGAN approach from pix2pix [3], uses spectral normalisation, and 64 filters in the last convolution layer, resulting in a receptive field of approximately 70×70 pixels. The GAN loss function is the Wasserstein loss function. For the generator, we also add ℓ_1 regularisation.

Data augmentation was performed on the fly during training using the Kornia [4] python module. Transformations included RandomGaussianNoise with a probability of 0.5 and a standard deviation of 0.04, RandomSharpness with a probability of 0.8 and a sharpness factor of 0.8, and RandomMedianBlur with a probability of 0.5 and a kernel size of 5×5 pixels.

3.2 Model training

All models were implemented in Pytorch Lightning and required on average 8 hours of training on a 4 x NVIDIA RTX3090. We trained three different models using different data splits, summarised below in table 2.

Table 2. Data splits used to create the various models. The mapping between scan number and geometry is listed in table 1.

Model name	Training scans	Validation scans	Test scans
Proton2Carbon_S	1,2,5,6,7	3,4	8, 9, 10, 11, 12
Proton2Carbon_G	5, 6, 7, 8, 9 10, 11,12	1, 2, 3	4
Proton2Carbon_A	1, 2, 8, 9, 10, 11, 12	3, 4	5, 6, 7

The model proton2Carbon_S was used to create the results of figure 2, 3, 6 as well as table 1 of the main manuscript. The model Proton2Carbon_G was used to generate the results of figure 4, while Proton2Carbon_A was used for figure 5.

References

[1] Simard, Mikaël, Ryan Fullarton, Lennart Volz, Christoph Schuy, Daniel G. Robertson, Allison Toltz, Colin Baker, Sam Beddar, Christian Graeff, and Charles-Antoine Collins Fekete. "A comparison of carbon ions versus protons for integrated mode ion imaging." Medical Physics (2024).

[2] Fullarton, Ryan, Mikaël Simard, Lennart Volz, Allison Toltz, Savanna Chung, Christoph Schuy, Daniel G. Robertson et al. "Imaging lung tumor motion using integrated-mode proton radiography—A phantom study towards tumor tracking in proton radiotherapy." Medical physics (2024).

[3] Isola, Phillip, Jun-Yan Zhu, Tinghui Zhou, and Alexei A. Efros. "Image-to-image translation with conditional adversarial networks." In Proceedings of the IEEE conference on computer vision and pattern recognition, pp. 1125-1134. 2017.

[4] Riba, Edgar, Dmytro Mishkin, Daniel Ponsa, Ethan Rublee, and Gary Bradski. "Kornia: an open source differentiable computer vision library for pytorch." In Proceedings of the IEEE/CVF Winter Conference on Applications of Computer Vision, pp. 3674-3683. 2020.
